# A Psycho-Educational HIV/STI Prevention Intervention for Internally Displaced Women in Leogane, Haiti: Results from a Non-Randomized Cohort Pilot Study

**DOI:** 10.1371/journal.pone.0089836

**Published:** 2014-02-28

**Authors:** Carmen H. Logie, CarolAnn Daniel, Peter A. Newman, James Weaver, Mona R. Loutfy

**Affiliations:** 1 Factor-Inwentash Faculty of Social Work, University of Toronto, Toronto, Canada; 2 Women's College Research Institute, University of Toronto, Toronto, Canada; 3 Faculty of Social Work, Adelphi University, New York, United States of America; University of New South Wales, Australia

## Abstract

**Background:**

Little evidence exists regarding efficacious HIV and sexually transmitted infections (STI) prevention interventions with internally displaced populations. Internally displaced women are at elevated risk for HIV/STI due to limited access to health services, heightened poverty and social network breakdown. The FASY (Famn an Aksyon Pou Sante' Yo) (Women Taking Action For Their Health) study examined the effectiveness of a peer health worker (PHW) delivered psycho-educational HIV/STI pilot study with internally displaced women in Leogane, Haiti.

**Method:**

This was a non-randomized cohort pilot study. Participants completed a computer-assisted pre-test programmed on Android tablet PCs followed by an HIV/STI educational video-based session and a 6-week psycho-educational group program of weekly meetings. Participants completed a post-test upon completion of group sessions. The primary outcome was HIV knowledge; our pre-specified index of clinically significant change was an effect size of 0.30. Secondary outcomes included: STI knowledge, condom use, social support, resilient coping, depression and relationship control. We used mixed-effects regression to calculate mean outcome pre-post score change. This study was registered (clinicaltrials.gov, NCT01492829).

**Results:**

Between January 1-April 30, 2012 we assigned 200 participants to the study. The majority of participants (n = 176, 88%) completed the study and were followed up at 8 weeks, finishing April 30, 2012. Adjusted for socio-demographic characteristics, HIV knowledge (β = 4.81; 95% CI 4.36–5.26), STI knowledge (β = 0.84; 95% CI 0.70–0.99), condom use (AOR = 4.05, 95% CI 1.86–8.83), and depression (β = −0.63, 95% CI −0.88–−0.39) scores showed statistically significant change post-intervention (p<0.05).

**Conclusions:**

This pilot study evaluated a PHW psycho-educational HIV/STI prevention intervention among internally displaced women in post-earthquake Haiti. Pilot studies are an important approach to understand feasibility and scientific impacts of HIV prevention strategies in disaster contexts. Study results may inform HIV prevention interventions among internally displaced women in Haiti and can be tested for applicability with internally displaced women globally.

ClinicalTrials.gov: Identifier NCT01492829, URL: http://clinicaltrials.gov/ct2/show/NCT01492829?term=logie&rank=1

## Introduction

Haiti has the highest HIV incidence in the Caribbean and the Americas, with an estimated 1 in 50 persons infected [1]. It is also the poorest country in the region. The January 12, 2012 earthquake resulted in the collapse of Haiti's social, health and economic infrastructure. Three and a half years following the earthquake 280 000 persons remain displaced from the homes they were living in prior to the earthquake [2]. These internally displaced persons live in tents or precarious makeshift shelters across 352 camp sites; over three-quarters of these sites lack basic protection monitoring and camp management support and half lack access to basic health services [2].

Internally displaced women are at elevated risk for HIV and sexually transmitted infections (STI) due to limited access to sexual and reproductive health services, heightened poverty and breakdown of social networks [3]–[4]. Poverty and gender equity exacerbated women's HIV infection risks in Haiti prior to the earthquake [5]. For example, young women had lower rates of HIV knowledge than young men [6] and they were also twice as likely to have an STI [7]. Pre-earthquake statistics indicated that over half of people living with HIV in Haiti lacked access to antiretroviral treatment [6]. In spite of the HIV prevalence in Haiti, UNAIDS [6] indicated that a small minority of Haitian women (8%) and men (5%) aged 15–49 reported having an HIV test in the past 12 months and knew their status. Researchers focused on Chlamydia and gonorrhea incidence in rural Haiti recommended that STI prevention encompass STI education and condom promotion [8].

There is a scarcity of literature regarding efficacious HIV prevention strategies in Haiti, particularly following the 2010 earthquake. The effectiveness of using community health workers (CHW) to provide HIV-related services to people living with HIV (PLHIV) in rural Haiti and linking this to primary care is well documented [5], [9], [10]. The feasibility of these or other HIV prevention approaches in post-earthquake Haiti, where access and affordability of health care is limited, is underexplored. There is no standard approach to HIV prevention with internally displaced persons (IDP). Global work with IDP has predominately focused on measuring HIV incidence, condom distribution and ARV administration [11], [12]. Culture, gender and contextual factors have largely not been integrated into these approaches [13].

Social ecological models that contextualize multi-level interactions between intrapersonal (e.g. knowledge, practices), interpersonal (e.g. social support, relationships with significant others), community (e.g. socio-cultural norms) and structural (e.g. advocacy, policy) factors that shape health outcomes have demonstrated some effectiveness [14]. Evidence from South Africa demonstrated that interventions that address social and structural drivers of HIV and STI were effective in reducing HIV/STI incidence and self- reported sexual risk behaviors [15]. Curriculum-based peer-led group interventions have also been effective in Sub-Saharan Africa in improving HIV knowledge [16] and positive social norms regarding HIV prevention [15], and increased condom use, partner communication and self-efficacy for safer sex [17].

We developed and pilot tested a peer health worker delivered intervention. Pilot testing was an appropriate approach for this study as there was limited information about approaches to effective gender-focused HIV prevention in post-disaster contexts, specifically in post-earthquake Haiti. Pilot testing also affords the opportunity to explore a project's feasibility—we were particularly interested in understanding recruitment and retention rates, assessing resources required, and examining scientific impacts of the intervention [18]. The information provided by this pilot-study will be used to inform the development of a larger randomized controlled trial for internally displaced women in Haiti.

We worked with peer health workers based on evidence that training and working with peers is effective in delivering and engaging communities in interventions and also promotes capacity building [5], [9], [10], [19]. Peer health workers (PHW) are a type of CHW that have been effective working in HIV interventions and providing psychosocial support in low resource settings [20]. A peer typically refers to a person with shared lived experiences as participants [20]; we refer to peers in this study as women who had the experience of being displaced from their homes in post-earthquake Haiti. Working with PHW is particularly salient in Haiti where there is a serious shortage of trained health care workers; Haiti was reported to have 2.5 doctors and 1.1 nurses per 10 000 persons [9]. Furthermore we built on previous literature that highlights efficacy of peer led group-based interventions that address social and structural factors [15]–[17]. The intervention was informed by social ecological theory and addressed intrapersonal (e.g. HIV and STI knowledge, coping), interpersonal (e.g. social support, safer sex negotiation), community (e.g. gender norms) and structural (e.g. community change) factors.

Leogane was the epicenter of Haiti's 2010 earthquake and is located approximately 30 km from Port-au-Prince. Leogane was devastated by the quake; 80–90% of its buildings were destroyed and approximately 20–30,000 people of out an estimated 120,000 population died [21]. As of June 2013 over 5,000 persons in Leogane remained displaced across 15 internally displaced persons sites [2].

The aim of the FASY (Famn an Aksyon Pou Sante' Yo) (Women Taking Action For Their Health) pilot study was to evaluate whether, compared with pre intervention, internally displaced women who receive the FASY individual and group psycho-educational HIV/STI prevention intervention in Leogane, Haiti, would demonstrate increased HIV knowledge scores. The secondary objective was to assess if, compared with pre intervention measures, internally displaced women who received the FASY intervention would demonstrate the following changes post intervention: increased STI knowledge scores; increased consistent condom use; increased social support; higher resilient coping; lower depression; and higher relationship control.

## Methods

The protocol for this trial and supporting TREND checklist are available as supporting information; see Table S1 and Protocol S1.

### Ethics Statement

Research Ethics Board approval (2011-0033-E) was obtained from Women's College Hospital, University of Toronto, Toronto, Canada.

### Study design and participants

FASY was a single-centre non-randomized cohort pilot study. The protocol for this trial has been published elsewhere [22]. This study was registered at clinicaltrials.gov, NCT01492829. We conducted this study between January 1, 2012–May 1, 2012: recruitment January 2012; pre-test February 2012; group sessions February–March 2012; post-test April–May 2012.

Internally displaced women (n = 8) were hired and trained as PHW and recruited participants in Leogane and contiguous areas. The collaborating agency, NEGES Foundation, purposively selected PHW who were internally displaced women with strong social networks; PHW were selected to be representative of a range of ages (21–60), diverse neighborhoods, and socio-economic backgrounds. Eligibility criteria for PHW included women over 18 years old with high rates of oral and written literacy in Kreyol. PHW were supervised by study coordinators at NEGES. This purposive selection strategy aimed to recruit PHW who would reflect and recruit diverse participants. To enhance confidentiality PHW received training on ethics and confidentiality, signed confidentiality agreements, and participants were guided to fill out private questions independently using color coded responses on the tablets.

We used modified purposive peer driven recruitment (PDR) methods. Peer driven recruitment is an effective strategy to engage marginalized populations in HIV research [23], [24]. Word-of-mouth and snowball sampling were used in conjunction with peer driven recruitment approaches. PHW verbally recruited participants from social networks, with the number of participants each PHW could recruit pre-determined (n = 25) [23]. Due to low literacy no print materials were used in recruitment. All recruitment and study components were conducted in Kreyol (local dialect). A local collaborating community development agency (NEGES Foundation) hosted the intervention: the study coordinator and assistants were based there and provided assistance with participant recruitment. Those who agreed to participate provided their first names and mobile phone numbers to the PHW.

The recommended sample size for linear multiple regression (R^2^ increase) with 7 predictors as calculated using G*Power 3.1 (effect size 0.15, p<0.05, power: 0.95) is 153 [25]. The pre-specified index of clinically significant change was an effect size of 0.30; the equivalent correlation for this effect size is 0.15, referring to a 15% difference in pretest and post-test scores. This effect size of 0.30 is equivalent to a small effect size based on Cohen's classification [26].

Eligible participants were women over 18 years old who were capable of providing informed consent and were internally displaced (living in a tent, camp and/or different location than prior to the 2010 earthquake) residing in Leogane or contiguous areas. To enhance comprehension of informed consent processes among participants with low literacy the informed consent documents were read aloud to participants and participants signed with an X. Persons who did not identify as a woman, internally displaced, were incapable of providing informed consent, and/or were not interested/able to attend the 6 week group-based program were excluded.

### Randomisation and masking

Randomisation and allocation were not relevant due to the non-randomized trial design. No blinding was undertaken as this was a pilot study that involved assessing the feasibility, study procedures and documents. The unit of assignment was individuals; as this was a non-randomized sample with no control group, all eligible participants were assigned to the intervention.

### Procedures

Participants in the intervention provided informed consent and completed a tablet-based pre-test survey assisted by the PHW at NEGES. Immediately following survey completion participants watched a brief (10 minute) HIV educational video in Kreyol on the tablet with the PHW. The video reinforced items on the HIV knowledge scale and the PHW were trained to answer any questions that arose during or after watching the video. Participants were provided with a $5 US honorarium for survey completion.

Following pre-test survey completion participants received 6 group-based psycho-educational sessions, each lasting 2–2.5 hours. Sessions took place at NEGES Foundation. The intervention was delivered to groups of 25 participants; each PHW led 6 weekly sessions with the same group of participants. PHW worked in pairs to provide additional support for the group sessions.

Sessions followed a manual that was developed based on content from the Population Council's ‘It's All in One Curriculum: A Unified Approach to Sexuality, Gender, HIV and Human Rights Education’ and included discussion points, information, case studies and activities to guide each session [27]. To enhance content validity an extensive process of pilot-testing each session and adapting content for the local context was undertaken, as well as translation to Kreyol and back-translation to English [22]. Weekly meetings addressed: 1) HIV and AIDS; 2) STI; 3) interpersonal relationships; 4) communication and decision-making skills; 5) mental health, resilience and coping and 6) creating community change. To enhance adherence participants were provided a small honorarium to compensate travel expenses, a snack and a beverage. If participants missed one session the PHW followed up with them to discuss barriers to their participation and brainstorm any solutions to facilitate attendance.

PHW (n = 8) and project staff (coordinator: n = 1; assistant: n = 1) received a 6-day training that involved basic research training (2 days) in study design, confidentiality, ethics, informed consent, recruitment, survey implementation, and group facilitation skills. The second component (4 days) involved training in the group session content area (HIV, STI, interpersonal relationships, communication, decision making, mental health, resilience, coping, community change). During the training each PHW took part as both a participant and a co-facilitator to demonstrate their knowledge of the content and experience delivering the intervention. The investigators (CHL, CD) conducted the training in Kreyol with a translator. The training was conducted in October 2011; project staff conducted a five-day refresher training with PHW (December 2011) and the investigators conducted a second 2-day refresher training (January 2012). Project staff supervised the first 5 pre-test surveys implemented by each PHW and monitored each group session to enhance fidelity of the intervention to the manual.

Participants completed the pre-test and watched the video in the 2 weeks prior to starting the 6-week program of group sessions. To reduce social desirability bias, questions pertaining to sexual risk practices were color-coded and the PHW were instructed on how to guide the participant to fill these questions out independently. Pre-test surveys included an identification number in order to compare pre and post-test scores. Identification numbers and corresponding participant name (first name, initial) were kept in a locked cabinet at NEGES. Post-tests were conducted in the 2 weeks following the completion of the 6 group sessions.

The survey was pilot-tested with service providers (n = 6) and the PHW (n = 8) to enhance clarity and relevance of measures to the Haitian context. This process involved reviewing survey items with the service providers and PHW for cultural, gender and contextual relevance for the target population of internally displaced women in Leogane. The primary outcome was HIV knowledge post-intervention, assessed using the Brief HIV Knowledge Questionnaire [28]. Secondary outcomes were post-intervention scores of: *STI knowledge*, measured using the Sexually Transmitted Disease Knowledge Questionnaire [29]; *relationship control* measured using the Sexual Relationship Power Scale's [30] ‘relationship control’ subscale; *condom use* assessed if participants consistently (‘always’) used condoms for sex in last 6 weeks with regular, casual and transactional sex partners; *depression* assessed using the Beck Depression Inventory Fast-Screen (BDI-FS) [31]; *resilient coping* measured using the Brief Resilient Coping Scale [32]; and *social support* from family, friends and a significant other using the Multidimensional Scale of Perceived Social Support (MSPSS) [33]. Several of these scales had been validated in resource-limited settings; for example the Brief HIV Knowledge Questionnaire in Nigeria [34], the BDI-FS and Brief Resilient Coping Scale in India [35], and the Sexual Relationship Power Scale [36] and MSPSS [37] in South Africa.

### Statistical analysis

Descriptive analyses of socio-demographic variables (e.g. age, income) were conducted. Descriptive statistics were calculated to determine frequencies, means and standard deviations (SD) for each variable. Scale, and where possible sub-scale, items were summed to calculate scores for outcome variables (HIV knowledge, STI knowledge, condom use, relationship power, depression, resilient coping, social support), except for condom use, which is dichotomous. Spearman's Rho correlational analyses, independent sample t-tests, and analysis of variance (ANOVA) were used to examine associations between socio-demographic and pre-intervention outcome variables. The analyses included all participants with available outcome data.

We used mixed-effects regression to calculate the pre-post outcome mean difference, and mixed-effects logistic regression to calculate post-intervention odds of condom use, adjusted for correlated structure of repeated measures data [38] while adjusting for baseline score socio-demographic differences. To calculate mean outcome pre-post score change we used mixed-effects regression, which accounts for clustering of 2 repeated measures (pre-intervention and post-intervention). This method uses maximum likelihood estimation for inference in order to allow inclusion of cases with missing data [39]. In addition to accounting for within- and between-subject variability, this method allows the flexibility to adjust for socio-demographic covariates.

For each outcome, we calculated pre-post outcome score change adjusted for socio-demographic covariates. For the single dichotomous outcome we report the odds of condom use at post-intervention similarly adjusted for socio-demographic characteristics. Because the study lacked a control group, we adjusted for all measured socio-demographic variables to avoid mistaking score change associated with socio-demographic profile as intervention effect. All analyses were conducted using STATA 11.2. Results will be made publicly available on clinicaltrials.gov, where the trial is registered.

## Results

**Figure 1:**
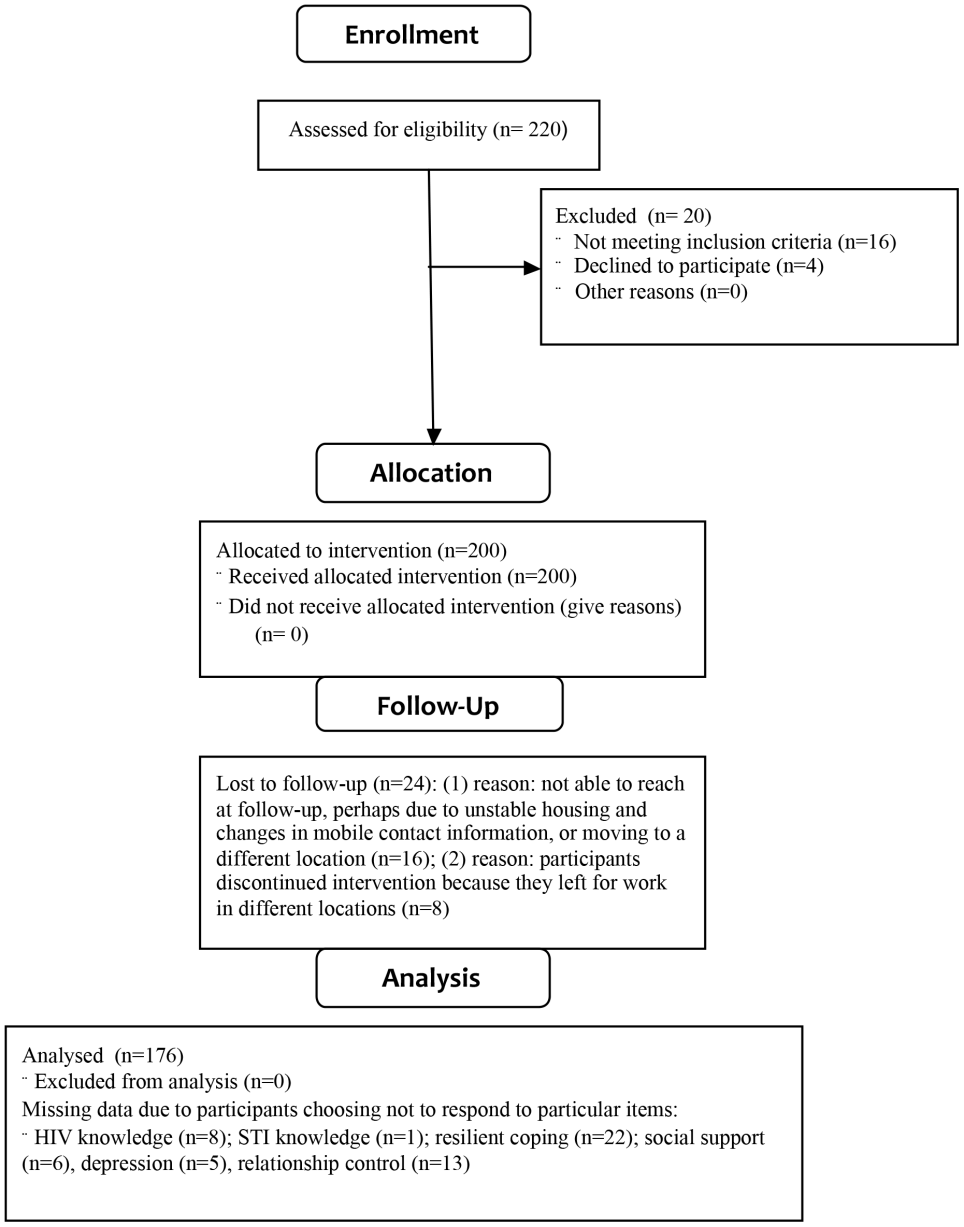
Flow chart of study.

As illustrated in Figure 1, there were 220 participants examined for eligibility; 200 were confirmed eligible and recruited for the study; 176 (88%) completed the group sessions, follow-up post-test, and were included in the analyses. Unstable housing and relocation could be reasons for participants lost to follow-up (n = 24). Participants were followed up within 2 weeks post intervention. Table 1 reports the socio-demographic characteristics of the sample. The majority of the 176 participants responded to pre-test outcome measures and post-test outcome measures; the number of responses per variable is reported in Table 2. Insufficient participants reported involvement in casual (n = 16), transactional (n = 14) or paid (n = 12) sex to include in condom use analysis. No adverse events or unintended effects associated with study participation were reported by project staff or participants.

**Table 1:** Socio-demographic characteristics of intervention participants (n = 176).

Variable	n	%
Age*	36.56	13.53
Education (missing = 1)		
Partial primary	58	33.14
Primary	54	30.86
Secondary	57	32.57
Partial uni./tech./prof.	6	3.43
Meals per day (missing = 1)		
0	22	12.57
1	102	58.29
2	47	26.86
3+	4	2.29
Income*	163.26	402.57
Marital status (missing = 1)		
Married	14	8
Living together	135	77.14
Dating – not cohabiting	21	12
Casual dating	5	2.86
Relationship duration (missing = 1)		
Less than 1 year	46	26.29
2–5 years	37	21.14
5–10 years	37	21.14
10+ years	55	31.43
Residence location (missing = 1)		
In town of Leogane	25	14.29
Outside of town	150	85.71
Number of children		
0	35	19.89
1 to 3	82	46.59
4+	59	33.52
HIV status (missing = 4)		
Positive	2	1.18
Negative	74	43.79
Unknown	93	55.03
Transactional sex (missing = 1)		
No	157	89.71
Yes	18	10.29
*mean (SD)		

**Table 2:** Observed pre- and post-outcome scores and adjusted pre-post differences.

Variables	Observed pre-intervention scores			Observed post-intervention scores			Adjusted mean difference[1]			
	n	Mean	SD	n	Mean	SD	Mean	95% CI		p
HIV knowledge	168	8.62	3.20	168	13.44	2.06	4.81	4.36	5.26	<0.001
STI knowledge	175	1.61	0.74	173	2.47	0.74	0.84	0.70	0.99	<0.001
Condom use[2]	176	0.16	0.37	176	0.32	0.47	4.05[3]	1.86	8.83	<0.001
Social support (SS)	170	36.47	7.68	175	37.81	8.09	1.02	−0.30	2.34	0.130
SS - significant other	174	13.30	2.99	175	13.57	2.94	0.17	−0.28	0.62	0.456
SS - family	171	11.51	3.51	175	11.98	3.59	0.45	−0.15	1.04	0.141
SS - friends	173	11.66	3.14	175	12.26	3.12	0.48	−0.09	1.06	0.100
Resilient coping	154	8.97	2.68	172	9.16	2.56	0.04	−0.36	0.45	0.837
Depression	171	3.31	1.60	173	2.68	1.08	−0.63	−0.88	−0.39	<0.001
Relationship control	163	40.29	6.41	173	40.96	4.93	0.43	−0.41	1.27	0.315

[1] Difference adjusted for age, education, meals per day, income, marital status, relationship duration, residence location, number of children, HIV status, transactional sex.

[2] Proportion always used condom.

[3] Adjusted odds ratio.

Participants were on average 36.6 years old and approximately one-third had partial primary (33.1%), one-third primary (30.9%), and one-third secondary school education or above (36.0%). The average monthly income was 163.3 Haitian Gourde (equivalent to $3.83 US) and the majority reported eating 1 meal or less per day (58.3%). Most participants lived with a partner (77.1%). Most women had 1–3 children (46.6%), compared to 4 or more (33.5%) and none (19.9%). Most women were of unknown HIV status (55.0%) or HIV negative (43.8%), whereas 2 (1.2%) were HIV positive. Approximately 10% engaged in transactional sex. There were no significant socio-demographic differences between participants lost to follow up and participants retained.

Pre-intervention primary and secondary outcome measure scores differed by certain socio-demographic characteristics: income, relationship status, relationship duration, location of residence, and number of children. Income was significantly and positively associated with HIV knowledge (*rs* = 0.263, p<0.05), STI knowledge (*rs* = 0.193, p<0.05), social support (*rs* = 0.152, p<0.05), resilient coping (*rs* = 0.198, p<0.05), and relationship control (*rs* = 0.187, p<0.05) scores. Participants who were married or cohabitating with their partner reported higher relationship control scores (F = 18.02, p<0.01) and lower depression scores (F = 4.22, p<0.01) than participants who were dating but not cohabitating. Relationship duration was associated with resilient coping: participants in relationships of 5 years or more had higher resilient coping scores in comparison with those in relationships shorter than a year (F = 4.39, p<0.01). Resilient coping scores were higher among participants who lived in Leogane in comparison with those who lived outside of town (OR: 1.36, 95% CI: 0.06, 2.66). Participants with children reported higher depression scores than those with no children (F = 3.14, p<0.05).

Table 2 reports observed main and secondary outcome pre- to post-intervention scores and the pre-post mean difference adjusted for socio-demographic covariates. For the single binary outcome measure, condom use, we report the observed pre and post proportions and the adjusted odds of condom use at post-intervention. The main outcome and 3 secondary outcomes showed change by post-intervention. Adjusted for socio-demographic characteristics, HIV knowledge (β = 4.81, 95% CI 4.36, 5.26), STI knowledge (β = 0.84, 95% CI 0.70, 0.99), condom use (AOR = 4.05, 95% CI 1.86, 8.83), and depression (β = −0.63, 95% CI −0.88, −0.39) scores showed statistically significant change by post-intervention at the p<0.05 level. The unadjusted effect size of time on the above outcomes were 0.67, 0.50, 0.18, 0.10, and 0.23, respectively.

## Discussion

A PHW-delivered intervention that involved a video-based individual session and a 6-week program of psycho-educational group sessions was effective in increasing HIV knowledge, STI knowledge, condom use, social support and in reducing depression among internally displaced women in Leogane, Haiti. Based on our pre-specified index of clinically significant change (ES: 0.30) we saw significant changes in HIV knowledge and STI knowledge. Participants included internally displaced women with very low income (<$4 US/month) and high food insecurity. The study completion rate was very high, with the majority of participants followed up post-intervention (88%). When socio-demographic variables and all outcomes were accounted for simultaneously, the majority of the outcome variance (58.9%) could be attributed to an effect from the intervention.

To our knowledge little published evidence exists regarding women-focused, peer-health worker delivered HIV/STI psycho-educational programs with samples of internally displaced women in post-earthquake Haiti. Thabane [18] described pilot studies as essential pre-requisites to randomized control trials and intervention scale-up. Hence, we believe that this pilot study provides valuable information about the feasibility of engaging internally displaced women as both PHW and participants in individual and group-based HIV/STI prevention strategies. Our results suggest that a social ecological approach that addresses intra/interpersonal, community and structural factors holds promise not only for increasing HIV and STI knowledge, and increasing consistent condom use, but also results in psycho-social benefits such as increased social support and reduced depression. Notably there were no significant changes in relationship control post-intervention, suggesting the importance of approaches that include male partners.

There are several limitations to this study, including the non-random sample and a lack of a control group, which reduce generalizability of the findings. The sample may be biased to include internally displaced women with greater social networks, resources, functioning, disability and health, which may overestimate the appropriateness of the intervention for internally displaced women with less networks or lower health outcomes. Due to our non-randomized study design we could not mask participants or researchers to treatment allocation. We did not have the financial or health resources available to provide HIV and STI testing and treatment, and therefore rely on self-reported risk behavior (e.g. condom use), which may be impacted by social desirability bias, and other indicators of HIV and STI vulnerability (e.g. HIV and STI knowledge). These limitations make extrapolation to different populations in and outside of Haiti difficult. Additionally, we only had one follow up point shortly following the intervention; thus we could not assess if the intervention effects were lasting or transient.

Future trials of HIV preventive interventions with this population would benefit by addressing these limitations. Obstacles to conducting this trial in post-earthquake Haiti were numerous, including limited access among participants to free HIV/STI testing and treatment, low rates of literacy, and unstable housing and relocation among participants. We addressed infrastructure challenges that were raised by participants, such as potential safety concerns due to lack of lighting at night, by adjusting the times the women's groups were held and ensuring that women did not walk home alone. We provided snacks before every meeting as food insecurity was a pressing concern. We also provided childcare for participants and permitted participants to bring babies and small children to the meetings. Future trials could also benefit from a) including a biological outcome such as HIV testing and linking the study to HIV testing and treatment, b) using a longitudinal design with multiple follow-up timepoints, accounting for potential relocation among study participants; and c) including internally displaced men in HIV/STI prevention initiatives, such as male-focused and couple-focused programs.

Despite these limitations we believe this study offers a strong theoretical foundation that can inform future randomized trials. We demonstrated the feasibility of hiring and training internally displaced women as PHW, and engaging internally displaced women in a 6 week program with a high retention rate (88%). While our median participant age was higher than the median age of Haitian women, there were similarities with fertility rates, poverty and unemployment in comparison with the general population of Haitian women [40]. This type of study may therefore be applicable to internally displaced women in other regions of Haiti. We did not find published reports of gender-specific, group based approaches to HIV prevention with similar populations (internally displaced persons) or in the region (Caribbean). We therefore compare our findings to psycho-educational approaches to HIV prevention in Sub-Saharan Africa. Interventions in the Sub-Saharan African context that addressed social factors (e.g. social support, gender norms) reported similar findings regarding reduced sexual risk behaviors (e.g. increased condom use) [15], [17] and increased HIV knowledge [16].

There are over 27 million internally displaced persons globally [41], and internally displaced women are particularly vulnerable to HIV/STI infection due to social and structural contexts of poverty, gender inequity and violence [3], [4]. Scant published research has explored HIV/STI prevention interventions that address these contexts. Approaches that integrate cultural, gender and contextual factors in HIV/STI preventive interventions with internally displaced persons—and engage internally displaced persons in the development and implementation of these interventions—warrant further attention [13]. The FASY approach, engaging internally displaced women as PHW and participants in an individual and group-based strategy, shows promise as such an intervention. Greater investment in engaging internally displaced persons in contextually appropriate HIV/STI interventions, and rigorous evaluation of these approaches, will provide the evidence base to support effective ways forward.

## Supporting Information

Protocol S1 **Development and evaluation of a tablet-based, community health worker delivered HIV/STI prevention intervention for women living in internally displaced persons camps in Leogane, Haiti.** The protocol is attached to outline the study: overview; background; objectives; research questions; methodology; research partnership; risks and benefits; timeline.
(DOC)

Table S1 **TREND Checklist.** The TREND statement checklist, designed to enhance reporting of trials with non-randomized designs, was completed to facilitate transparent reporting of this intervention.
(DOC)
