# Supplementary material for: A Psycho-Educational HIV/STI Prevention Intervention for Internally Displaced Women in Leogane, Haiti: Results from a Non-Randomized Cohort Pilot Study
Source: PLoS One. 2014 Feb 28;9(2):e89836. doi: 10.1371/journal.pone.0089836 (PMC3938530; doi:10.1371/journal.pone.0089836)
Supplement: Protocol S1 — Development and evaluation of a tablet-based, community health worker delivered HIV/STI prevention intervention for women living in internally displaced persons camps in Leogane, Haiti. The protocol is attached to outline the study: overview; background; objectives; research questions; methodology; research partnership; risks and benefits; timeline. (DOC) [file pone.0089836.s001.doc]

**Protocol S1.** Development and evaluation of a tablet-based, community health worker delivered HIV/STI prevention intervention for women living in internally displaced persons camps in Leogane, Haiti.

**Principal Investigator**: Dr. Carmen Logie, Women’s College Research Institute

**Co-Investigator:** Dr. Carol Ann Daniel, NEGES Foundation/Adelphi University

**Funding Agency:** Grand Challenges Canada Rising Star in Global Health

**1. OVERVIEW**

We will develop, deliver and evaluate a solar powered tablet-based motivational interviewing HIV/Sexually Transmitted Infection (STI) intervention at internally displaced persons (IDP) camps in Leogane, Haiti. Women from IDP camps will be trained as community health workers to deliver the HIV/STI intervention to women in IDP camps working in collaboration with NEGES, a community development organization.

**2. BACKGROUND**

Haiti has the highest HIV infection rate in the Western Hemisphere, with 1 in 50 people infected (UNAIDS, 2011). Almost half (46%) of people living with HIV (PLHIV) in the Caribbean live in Haiti (UNAIDS 2011b). The January 2010 earthquake led to the collapse of Haiti’s social, economic and health infrastructure. One year following the earthquake, UNICEF (2011) reported

that one million people remain displaced, living in approximately 1,200 IDP camps. UNICEF’s January 2011 report noted that there is currently no organization responsible for community based HIV prevention, condom distribution or HIV/AIDS education in IDP camps in Haiti.

Women who live in IDP camps are at particularly high risk for HIV infection and sexually transmitted infections (STI) due to limited access to reproductive health services, the collapse of social infrastructure and community networks, increased poverty and sexual violence (IASC, 2010; MADRE, 2011; UNAIDS, 2011b).

It is integral to involve women’s grassroots organizations in the planning and delivery of HIV prevention interventions and strategies to reduce sexual violence to ensure their success (MADRE, 2011). There is an urgent need for more information on HIV risk and prevention in the context of violence among women in Haiti’s IDP camps (UN Women, 2011).

The deleterious impact of HIV and AIDS is exacerbated due to a weak health care system, extreme poverty and HIV-related stigma (UNAIDS, 2010). Haiti is the poorest country in the Western hemisphere: in 2009 it ranked 145th out of 169 countries on the United Nations Development Programme’s Human Development Index. Poverty and a lack of employment opportunities pose serious barriers to health and well-being. In Haiti less than half (43%) of

PLHIV have access to anti-retroviral treatment, and only 60% of HIV-positive women have access to anti-retrovirals (ARV) for preventing perinatal HIV transmission (UNAIDS, 2010).

Even before Haiti’s 2010 earthquake, women and girls were at higher risk for HIV infection. Young women aged 15-24 have HIV infection rates double those among young men (UNAIDS, 2010). Knowledge regarding HIV/AIDS is very low in Haiti: approximately one-third of young men and women aged 15-24 could correctly identify HIV prevention methods and reject major HIV transmission misconceptions (UNAIDS, 2010). Young women had lower knowledge rates

(34%) than young men (40%). More women (79%) than men (66%) aged 15-49 who had more than one sexual partner in the past 12 months did not report condom use during their last sexual encounter (UNAIDS, 2010). Only 8% of women and 5% of men aged 15-49 reported receiving an HIV test in the last 12 months and knowing their status (UNAIDS, 2010). Research with youth

(n=3391) in Port-au-Prince reported HIV infection was associated with: lower levels of education, STI infection, having a child, not living with one’s parents; this study also found that most youth had never used a condom (Dorjgochoo et al., 2009). Authors highlighted an acute need for HIV prevention services targeting youth, with a particular focus on building self-esteem and sexual negotiating skills among girls and young women (Dorjgochoo et al., 2009).

Developing a women-focused community-based model of HIV prevention in IDP camps in Haiti has implications for Canada’s HIV prevention work globally. There are over one million IDP in Haiti, and over 27 million IDP globally (IDMC, 2011): this study can therefore inform HIV prevention research/programming in Haiti and can be tested for applicability in other IDP camps globally. Researchers have highlighted the need for creative partnerships to share skills and HIV

care models between developed and developing countries (Ivers et al., 2010). Struggles to meet basic needs for survival and sexual violence have been highlighted as drivers of HIV among IDP in Uganda (Rujumba & Kwiringa, 2010), so lessons learned may inform global HIV prevention.

Global HIV work in IDP camps has focused on measuring HIV incidence, distributing condoms, or ARV distribution (Hampton, 2008; Khaw et al., 2000)—less research has focused on cultural, gender and context appropriate HIV prevention strategies for women (Spiegel, 2004).

**3. OBJECTIVES**

This study aims to:

1. Understand levels of: HIV/STI knowledge and risk factors; coping (social support, resilient coping); mental health issues (trauma, depression); and relationship power
2. Assess the influence of individual and group educational sessions on: HIV/STI knowledge and risk; coping (social support, resilient coping); mental health issues (trauma, depression); relationship power
3. Conduct a community forum with partners, health researchers and knowledge users in Leogane, Haiti to share HIV/AIDS information and study findings

**4. RESEARCH QUESTIONS**

1. What are the levels of: HIV/STI knowledge, HIV/STI risk factors, mental health issues, coping, and relationship power among women living in IDP camps in Leogane, Haiti?
2. Holding all other effects constant, is there a significant difference in outcomes (HIV/STI knowledge, HIV/STI risk factors, mental health issues, coping, relationship power) among women living in IDP camps before and after a peer health worker delivered HIV/STI intervention?

**5. METHODOLOGY**

**5.1 Overall approach**

We will develop and field test a solar-powered tablet technology that synchronizes HIV/STI prevention messages with a participant’s responses in Leogane, Haiti. The tablet will be programmed to analyze the data and to generate a brief video-based counseling message for the peer health worker (PHW) and participant that involves HIV/STI risk reduction strategies.

A pre and post-test N-of-1 trial design will be used. Outcomes (HIV/STI knowledge, HIV/STI risk factors, mental health issues, coping, relationship power) will be measured at baseline and 3 months post-intervention. Participants (n=200) will receive a high intensity HIV/STI educational intervention: a) tablet-based intervention using an HIV/AIDS educational video; b) 6 weekly sessions of women’s health discussions. The control group will include the same group by testing the outcomes at baseline.

**5.2 Inclusion/Exclusion Criteria**

**Research participants** include women living in IDP camps in Leogane, Haiti. The participants will be recruited by trained peer health workers (PHW), NEGES community development agency and other community organizations serving women in Leogane, Haiti. We aim to recruit a total sample of 200 women.

Inclusion criteria for research participants include adults over 18 years old who are: capable of providing informed consent; living in an internally displaced persons camp; interested in attending the 6-week program of women’s health discussions; interested in conducting a post-test survey.

**Peer health workers** will include 8 women living in IDP camps in Leogane, Haiti who have high rates of literacy in oral and written Haitian Creole and experience conducting research and/or educational activities. Dr. Carol Ann Daniel, program development advisor at NEGES Foundation, will identify women interested in being hired as peer health workers (PHW). PHW will be supervised by the study coordinator based at NEGES and Dr. Carol Ann Daniel.

**5.3 Study components**

There are 6 components to the overall project:

(1) developing a survey to assess outcomes (HIV/STI knowledge and risk factors, trauma, depression, resilient coping, social support, relationship power)

(2) developing a peer health worker training manual and HIV educational videos

(3) training women in IDP camps in Leogane, Haiti to be peer health workers (PHW)

(4) conducting tablet-based pre-test and post-test surveys to collect information on outcomes among women living in IDP camps in Leogane, Haiti

(5) conducting 6 weekly sessions with women living in IDP camps in Leogane, Haiti

(6) conducting a community forum including women living in IDP camps to share basic HIV/STI information and to disseminate findings from the project

**5.3.1 Activities:**

***(1) Developing a survey to assess outcome measures/goals***

Previous literature, outlined in the Introduction section, has highlighted high rates of HIV and STI and low HIV/STI knowledge among women in Haiti. The survey will be based on pre-existing scales with established reliability and validity (*described in ‘outcome measures’ section below*) will be translated into Haitian Creole. We will hold two focus groups to pilot test scales. Focus groups will be held with: service providers (n=1) and peer health workers (n=1) in Leogane, Haiti to assess outcome measures. Each focus group will include 6-10 participants, will be conducted in Haitian Creole, transcribed, translated into English and analyzed using thematic analysis (Braun & Clark, 2006). Focus groups will involve reviewing each outcome measure for its cultural, gender and contextual relevance for women in Leogane. Surveys will be modified based on focus group feedback to ensure culture, gender and context appropriateness. Participation in pilot testing will be voluntary. There will be no repercussions for those people who choose not to pilot test the tool. This feedback will be integrated into the development of the final survey. Results from pilot testing will not be included in data analysis and surveys will be destroyed immediately.

***(2) Develop a training manual and educational video messages***

A training manual will be developed for the PHW: this training manual will include discussion points, information, and activities to guide each of the weekly women’s health meetings. The training will be primarily based on the Population Council’s ‘It’s All ONE Curriculum: A Unified Approach to Sexuality, Gender, HIV and Human Rights Education’ (http://www.popcouncil.org/publications/books/2010_ItsAllOne_Who.asp). The ‘It's All One Curriculum’ is designed primarily for community educators responsible for education in the areas of sexuality/sexual health. The Population Council describes with this curriculum: *“Users may draw on the guidelines and activities in this kit to meet their needs, for example: to develop or modify comprehensive curricula (of any duration) appropriate for their setting; to design more narrowly focused teaching units (for example, on gender or sexual health); and to use as a resource for single-topic lesson plans (for example, gender and the media, deciding about sex, protecting oneself and one’s partners from HIV, reflecting on masculinity).”* (Downloaded from: http://www.popcouncil.org/publications/books/2010_ItsAllOne_Who.asp)

This curriculum has been developed cross-culturally and provides case studies from around the world and provides guidance on how to adapt the materials for use in different contexts. The PI, co-investigator and coordinator will work collaboratively to ensure the sections developed for use in the training manual for this research project are culturally and gender relevant for women living in IDP camp settings in Leogane. Content areas for ‘It's All One Curriculum’ include: sexual health and well-being; gender; sexuality; interpersonal relationships; communication and decision-making skills; sexual and reproductive health.

***(3) Train peer health workers***

Peer health workers (PHW) will be considered employees and paid a rate equivalent to the hourly wage paid by the NEGES ($5/hour CAD; hired for 6 months for November to May at 20 hours/week). We will hire between 8-10 PHW who will be hired for training, participant recruitment, pre/post-test survey administration, and facilitation of weekly women’s health meetings. We will provide PHW with phone cards as needed to use for the duration of recruitment, survey implementation and weekly meetings. These phone numbers can be used by potential participants to contact the PHW, and for the PHW to contact the coordinator who will be based out of NEGES.

PHW will be considered members of the research team and their feedback and recommendations will guide recruitment strategies and survey implementation. The PHW will also have the opportunity to provide feedback on the survey itself through pilot-testing during the training.

In collaboration with NEGES we will develop and deliver a 10-day (paid) training that will consist of 2 components. The first component (2 days) will include basic research training: study design; confidentiality; ethics; informed consent; recruitment; survey implementation; facilitation skills for weekly meetings.

The second component (4 days) will involve training the PHW in the content areas for the six weekly women’s health meetings:

1. sexual health: HIV/AIDS
2. sexual health: sexually transmitted infections
3. interpersonal relationships
4. communication and decision-making skills
5. mental health and well-being
6. coping skills and social support

Content areas will be largely based on Population Council’s ‘It’s All ONE Curriculum’ (<http://www.popcouncil.org/publications/books/2010_ItsAllOne_Who.asp>). The content, discussion questions and activities will be implemented with the PHW who will have a chance to ask any questions and discuss any concerns. We will train the PHW for each weekly health session topic.

We will have the training documents translated into Haitian Creole; the PI, co-investigator (CD) and coordinator in conjunction with a translator will conduct the training at a private room in NEGES in Haitian Creole. Team debriefing will be provided on a bi-weekly basis by the coordinator based at NEGES and as needed.

The study coordinator will be hired to assist in: recruiting the PHW, acquiring project space and materials, developing the survey, recruiting participants, training the PHW, supervising community health workers, managing data collection, communicating with principal investigator and co-investigator, organizing the community forum, and co-facilitating the weekly women's health meetings at NEGES. The study coordinator will be based in Leogane, fluent in both English and Haitian Creole, familiar with Leogane and local community agencies, and experienced in supervising staff. The coordinator will be hired as part of the study team and paid $15/hour to work 20 hours/week for the study duration (18 months).

***(4) Conduct pre-test and post-test survey***

We will use a cross-sectional structured survey programmed on a tablet verbally administered by peer health workers. Eight peer health workers (PHW) will be hired: each PHW will administer the pre/post test to 25 participants. Benefits of verbally administered surveys include: enhancing accessibility to participants with differing levels of literacy; building trust between the interviewer and participant; and higher response rates (Mertens, 2005). I will provide training and written instructions to standardize and manualize the structured interviews. At the beginning of the interview the PHW will describe the sections that will be covered. Due to the sensitive nature of the topic (e.g. sexual risk factors) participants will be informed that they have the option to fill out the tablet-based survey (either the entire survey, or certain sections) independently if they wish and the peer health worker (PHW) will guide the participant on which button on the tablet to press to respond (e.g. top, middle, bottom) and will be present to answer any questions. It will be stressed to participants that their decision to participate is voluntary and they can withdraw from the study at any time. Participants can also skip questions or entire sections.

Following completion of the tablet-based survey, the tablet will be programmed to have an HIV/AIDS educational brief video (in Haitian Creole) on the tablet. The participant and PHW can watch the brief video (focused on HIV/STI education and prevention) together and the PHW will answer any questions the participant may have. Watching the video is optional and voluntary and the participant will be informed of this at the beginning of the survey and after completing the survey. Participants will be provided with an honorarium for the pre-test and post-test (equivalent to $5 CAD for each test). Participants will still be paid the full honorarium if they decide to not fully complete the survey or to leave when the survey is partially complete.

Surveys will be implemented in a private room within NEGES, an agency which participants are familiar with and many already attain services. Multiple strategies will be employed to protect confidentiality of participants. The PHW will be trained and 1) a significant part of the training will be regarding confidentiality and ethics and 2) the PHW will sign confidentiality agreements to protect confidentially of participants. No names or other identifying information will be collected on surveys or informed consent sheets, and informed consent sheets and surveys will be kept at a locked filing cabinet in NEGES. The surveys will have an ID number so pre and post-test scores can be compared. A sheet with the ID number and participants first name and last initial will be kept in a locked filing cabinet at NEGES; PHW will only have access to this sheet during data collection. Participants will also have an option to keep an informed consent sheet, which will be evidence of survey participation, or to have access to it at NEGES. Signatures will not be required for the informed consent; to enhance confidentiality participants can sign with an X.

***(5) Conducting weekly women’s health meetings***

Each PHW (n=8) will conduct a 2-hour women’s health meeting at NEGES once a week for 6 weeks. Meetings will be held when NEGES is closed and each PHW will conduct meetings at different times: this will help to ensure privacy. Each PHW will carry out the 6 meetings with the 25 women they conducted the pre test with. Participants will receive an honorarium for each session attended and food and refreshments will be provided. PHW will have a training manual with the outline for each session, including: learning points, a case study, activities and discussion questions. Each session will begin with ground-rules and discussions about confidentiality (e.g. importance of not sharing individual’s comments from the discussion outside of the meeting).

Weekly meetings will address: (1) sexual health: HIV/AIDS; (2) sexual health: sexually transmitted infections; (3) interpersonal relationships; (4) communication and decision-making skills; (5) mental health and well-being; (6) coping skills. Limits of confidentiality will also be discussed with participants, in particular participants will learn that confidentiality cannot be guaranteed during group meetings and to keep this in mind when they are sharing personal information. Sessions will be conducted in Haitian Creole, and PHW will record notes following each session regarding any questions or points that need clarification. The coordinator will meet with the PHW weekly to provide support, clarification and any guidance requested.

***(6) Conduct community forum***

A community forum will be held at NEGES in Leogane convening women, men and adolescents living in IDP camps, social/health care providers, community development workers, researchers and other stakeholders. This community forum will elicit community and professional perspectives on the research findings in order to identify priorities and strategies for future women-focused HIV research and programs living in IDP camps in Leogane. Community forums that bring researchers and stakeholders together can foster community capacity and partnerships.

This forum will: provide basic HIV/AIDS information; conduct member checking by sharing and discussing the findings; and engage community members in a dialogue regarding priorities for community-based HIV research and prevention programs. A report highlighting the findings regarding HIV/STI knowledge, mental health, relationship power, and coping will be prepared and translated into French and Haitian Creole for dissemination to community members and stakeholders. HIV/STI knowledge questions that emerge within the weekly discussions will also be addressed in this community forum: HIV/AIDS and STI informational fact sheets regarding transmission, treatment and care will be disseminated at the forum. The UNAIDS IASC (2010) highlighted that raising HIV awareness and fostering community support is an integral first step to addressing HIV in humanitarian settings.

**5.4 Outcome Measures**

The survey will be based on pre-existing scales with reported reliability and validity. The primary outcome is: (1) HIV knowledge. The secondary outcomes are: (2) STI knowledge, (3) condom use, (4) relationship control, (5) depression, (6) resilient coping, and (7) social support.

**5.4.1 Primary Outcome**

**HIV knowledge** will be assessed using the 18-item Brief HIV Knowledge Questionnaire (Carey & Schroder, 2002).

**5.4.2 Secondary Outcomes**

**STI knowledge** will be assessed using the Sexually Transmitted Disease Knowledge Questionnaire (Jaworski & Carey, 2007). **Relationship power** will be measured using the Sexual Relationship Power Scale (Pulerwitz, Gortmaker & DeJong, 2000). **Condom use** will be assessed using using 12 items that assess consistent condom use with regular, casual, transactional and paid sex partners. The Beck Depression Inventory for Primary Care (BDI-PC), also known as the Beck Depression Inventory Fast-Screen (BDI-FS) is a seven item **depression** screening tool developed to provide a quick assessment that focuses on non-somatic items associated with depression and has a quick completion time (estimated 5 minutes) (Beck et al., 1997). The original BDI-FS includes an item on suicidality that will not be included in this survey as the PHW may not feel adequately trained to cope effectively with a suicidal participant and may not have the resources to provide appropriate crisis management. Golden et al. (2007) reported that omitting the suicidality item did not impact its reliability and may in fact increase the acceptability of this tool to participants. **Resilient coping** will be assessed using the 4-item Brief Resilient Coping Scale (Sinclair & Wallston, 2004). The 12-item **Multi-dimensional Scale of Perceived Social Support** (Zimet, Dahlem, Zimet & Farley, 1988) will be used to measure social support.

**5.5 Data Collection and Analysis**

The surveys will be administered in Haitian Creole, the primary language in Haiti. The survey will be developed in English, translated into Haitian Creole, back-translated into English, and finalized in Haitian Creole. The surveys will be programmed into a tablet; each PHW will be assigned one tablet for the duration of the study. Once the PHW have completed each survey, they will return the tablet to a locked filing cabinet at NEGES. The survey results will be entered into the password-protected tablet. Following the survey, the PHW will save the data from the tablet on a password protected, encrypted USB key in the locked filing cabinet in NEGES. Each PHW will be assigned their own: USB key (password protected, encrypted), tablet, and codesheet. Only the study coordinator will have access to the locked filing cabinet at NEGES.

The principal investigator will travel to Haiti during the pre (January 2012) and post-test (April 2012) data collection. After data collection is completed for the pre-test, the data will be removed by the principal investigator from the tablets and USB keys and stored on a password protected laptop password-protected computer, specifically into an SPSS database. After data collection is completed for the post-test, the data will be removed by the principal investigator from the tablets and USB keys and stored on a password protected laptop password-protected computer, specifically into an SPSS database.

**5.6** **Participant Recruitment**

**5.6.1 Sample Size Justification**

This study will aim to recruit 200 participants. There are 7 outcome variables, and a minimum sample size of 20 participants per variable is recommended for MANOVA (Field, 2009). Due to the possibility of attrition in this population we will aim to recruit 200 participants although 160 would be the minimum sample size required to detect a significant effect.

**5.6.2** **Sampling Methods**

Survey participants will be recruited through trained peer health workers (PHW) hired for the research study as well as a study coordinator hired and based at NEGES community development agency. Purposive sampling methods will be used, specifically a) venue-based and b) peer-driven recruitment. Many potential participants are illiterate and do not have telephones; thus no recruitment will be done from participants’ homes and no flyers or print advertising will be used.

**Peer-driven Recruitment:** PHW will verbally disseminate information about the study and invite eligible participants, using a peer driven recruitment (PDR) technique. Peer driven sampling techniques are a component of participatory action research and involve: direct contact between the PHW and the participant, recruitment from social networks, and regulating the number of participants each PHW can recruit (Magnani, 2005). For this research project, each PHW can recruit a maximum of 25 participants. Peer to peer recruiting has been found to be an effective strategy to engage marginalized and stigmatized populations in HIV research and has been found to have positive effects with regard to informal peer education, health promotion and sharing of HIV resources (Tiffany, 2006). PDR can also be used to disseminate research findings back to marginalized populations.

As described by Tiffany (2006), we will follow the steps of PDR: 1) engaging stakeholders (i.e. NEGES) in planning this research, 2) engaging with PHW recommended by community organizations (e.g. NEGES, 3) involving PHW in planning the recruitment strategy and pilot testing the survey, 4) employing PHW to recruit participants who may then choose to inform people within their social networks of the study. Special care is taken in the PHW training to explain informed consent, confidentiality and voluntary participation to PHW due to this sampling technique. PHW will sign confidentiality agreements. The peer driven recruitment approach “to recruiting members of hidden, vulnerable and marginalized populations to participate in research projects can contribute to more inclusive community dialogue about social issues and strengthen community participation in research efforts” (Tiffany, 2006, p. 122).

PHW are not actively engaged in service provision within NEGES or any community agencies related to the research. There may be a personal relationship (i.e. friendship) between some participants and PHW, as the PHW will be involved in peer-driven recruitment. PHW will be assigned to work with participants they do not have close personal relationships with. Participants can be guided to complete the tablet-based survey privately to maintain confidentiality and privacy. PHW have previous research experience in conducting recruitment, research and/or education and will undergo training regarding ethics, confidentiality, recruitment and informed consent; it will be stressed to participants that their decision to participate is voluntary and they can withdraw from the study at any time. As potential participants may be illiterate and may not have phones, no flyers or print advertising will be used. All recruitment activities will be conducted in Haitian Creole. The study coordinator will help with recruitment and support the PHW in the recruitment process.

**5.7 Informed Consent Process**

To assess if participants are capable of providing informed consent the following strategy will be used. The informed consent process will take place in a private room at NEGES. The PHW will read aloud the informed consent in Haitian Creole, while the participant follows along, if she is capable, reading silently. The PHW will pause and ask the participant if they have any questions about what has just been read. After reading the informed consent completely, the PHW will ask if the participant has any questions about anything they have read. If there are any questions, the PHW will answer them. Then, the PHW will ask a few questions about the procedures explained in the informed consent. If the participant cannot answer the questions or appears to be confused, the PHW will review the involved section of the informed consent again. If the participant cannot answer the questions at this time, they will be notified by the PHW that they don’t meet the inclusion criteria, they will be paid the full honorarium for the pre or post-test and will be excluded from the study. If the PHW assesses that they are upset, they will be referred to an intake worker at NEGES who will provide counseling and referrals to appropriate supports and community resources.

Participants will be asked to mark X in a box on the consent form to indicate their consent and the PHW will sign the consent form in front of the participant. As the greatest risk to confidentiality is participant names on consent forms, and because some participants may be illiterate, the X will be used to ensure confidentiality. Additionally, in order to avoid any risk of disclosure of study participation as a result of participants’ retaining a copy of the informed consent form, an unsigned copy of the consent form will be maintained on file at NEGES for participants to view if they wish, as explained in the consent form. Furthermore, the informed consent process will explicitly state that participation or non-participation in the study will no way affect eligibility for and receipt of services from NEGES or any other agency.

It will be stressed to participants that their decision to participate is voluntary and they can withdraw from the study at any time. Participants can also skip questions or entire sections. Participants will still be paid the full honorarium if they decide to not fully complete the survey or to leave when the survey is partially complete.

There will be no personally identifiable information collected. The first question on the tablet will be regarding an ID#. A sheet with ID# and initials (first name, last initial) will be kept in a locked filing cabinet at NEGES and only accessed by the PHW before they conduct a survey to ensure the same ID# is used for participants in the pre and post-test. This sheet will be destroyed after the post-test data is collected.

To mitigate any difficulties in understanding the informed consent process, the PHW will be trained on providing informed consent verbally to participants. It will be stressed to participants that their decision to participate is voluntary and they can withdraw from the study at any time. I will also pilot test the survey with the PHW, make any clarifications to the survey and informed consent that are recommended. Also, the coordinator will be based at NEGES and will meet with the PHW regularly to debrief with them about the process and any support they need in implementing the surveys and/or weekly meetings.

**5.8 Data Analysis**

**5.8.1 Descriptive Statistics**

Descriptive analyses of socio-demographic (e.g., age, income) variables, including means and standard deviations, will be conducted to provide an overview of participant characteristics. Secondly, items for each scale will be summed to calculate scores for HIV knowledge, STI knowledge, HIV/STI risk factors, relationship power, depression, trauma, resilient coping, and social support. Descriptive statistics will be calculated to determine frequencies, means and standard deviations for each summed score. I will utilize SPSS to conduct my analysis.

**5.8.2 MANOVA Analyses**

Multivariate analysis of variance (MANOVA) will be used to assess pre and post-test differences across outcome variables. Separate MANOVA analyses will be conducted for primary outcome variables focused on HIV/STI (HIV knowledge, STI knowledge, HIV/STI risk factors, relationship power) and secondary outcome variables focused on mental health (depression, trauma, resilient coping, social support). We will compare differences in participant scores from the pre-test and post-test (after the 6-weekly health meetings) to assess if the intervention influenced any of the primary or secondary outcomes. We will conduct a multivariate analysis of covariance (MANCOVA) to control for the number of weekly health meetings attended. Significant MANCOVA will be followed up with both univariate tests and discriminant function analyses (DFA) to understand significant effects. DFA can be used to identify variates (combinations of dependent variables) and how the dependent variables contribute to the variates. Analyses will be conducted using SPSS 19 software.

**6. RESEARCH PARTNERSHIP**

This research project is a partnership between a social science researcher (PI: Carmen Logie) at Women’s College Hospital and a community development practitioner/researcher at NEGES and Adelphi University (Co-investigator: Carol Ann Daniel). Founded in 1997, NEGES is a community development organization located in Leogane, Haiti. NEGES provides services to improve health and well-being and to promote capacity building.

This project builds on—and strengthens—relationships among HIV researchers focused on African and Caribbean women’s health. C. Logie is a Canadian Institutes of Health Research Post-doctoral Fellow in the Women and HIV Research Program at Women’s College Research Institute, Women’s College Hospital. C. Logie has expertise in international HIV research, stigma and discrimination, and research with African Caribbean women. Specifically, C. Logie has five years of national and international community-based research experience. Her international experience includes: her doctoral research conducted with sexual minorities in South India; coordinating a research project with female sex workers in Thailand; conducting national HIV prevention programs and research with youth in Ghana. Her national research experience includes: her post-doctoral research focused on stigma, discrimination and health among diverse HIV-positive women in Ontario; coordinating research projects involving diverse communities including African Caribbean women in Ontario; and assisting on social work research focused on cultural competence.

C.A. Daniel is an Associate Professor at Adelphi University, New York and a Program Development Advisor with NEGES. C.A. Daniel has expertise regarding Caribbean women, cross-cultural research, trauma, and field experience in Haiti. Specifically, C.A. Daniel has been working with NEGES to develop projects to promote mental health among women living in IDP camps in Leogane, Haiti, since the 2010 earthquake. C. Logie has worked with C.A. Daniel on HIV research with Caribbean women since 2009. C. Logie met with C.A. Daniel and other NEGES staff in October 2010 and February 2011 to discuss conducting HIV research in Leogane. NEGES staff highlighted the need to conduct HIV research and prevention programs for women living in IDP camps in Leogane. The study design for this project was developed in collaboration with C.A. Daniel and NEGES staff in February, 2011.

**7. RISKS AND BENEFITS OF PROPOSED RESEARCH**

**7.1 Risks**

**Psychological/emotional risks** include that participants may feel uncomfortable, anxious or upset answering questions about HIV, STI, sexual risk factors, depression, trauma, and relationship power. These risks will be mitigated by ensuring the study population includes adults deemed capable of providing informed consent by the trained peer health worker (PHW). The process of determining ability to provide informed consent includes having the PHW read aloud the informed consent in Haitian Creole, while the participant follows along, if she is capable, reading silently. The PHW will pause and ask the participant if they have any questions about what has just been read. After reading the informed consent completely, the PHW will ask if the participant has any questions about anything they have read. If there are any questions, the PHW will answer them. Then, the PHW will ask a few questions about the procedures explained in the informed consent. If the participant cannot answer the questions or appears to be confused, the PHW will review the involved section of the informed consent again. If the participant cannot answer the questions at this time, they will be notified by the PHW that they don’t meet the inclusion criteria, they will be paid the full honorarium and will be excluded from the study. If the PHW assesses that they are upset, they will be referred to an intake worker at NEGES who will provide counseling and referrals to appropriate supports and community resources.

Also, since participants have been recruited using a peer driven approach, participants may already have connections with peers and/or community development and/or health organizations thus may be conversant in these topics.

In order to minimize psychological/emotional risks about feeling uncomfortable, anxious or upset with study questions:

1. Participants may skip any questions if they feel uncomfortable or do not wish to answer

2. It will be stressed to participants that their decision to participate is voluntary and they can withdraw from the study at any time, with no adverse consequences, and will still be paid the full honorarium

3. Participants may fill out the tablet-based survey themselves instead of having it verbally administered by the PHW. The PHW can provide verbal directions and guidance to assist the participant to answer the questions on the tablet privately.

4. The Beck Depression Inventory –Fast Screen (BDI-FS) will be used, however only 6 items will be used. The item on suicidality will not be included as PHW may not feel adequately trained to cope effectively with a suicidal participant and may not have the resources to provide appropriate crisis management.

5. Participants will be provided with a list of community resources, including community health and mental health agencies and government run clinics. Participants will also be provided a contact number for NEGES of an individual who can provide support and services who is not a member of the research team. Participating or not in the study in no way affects access to services at NEGES or any other community agency.

**Social risks** include loss of privacy and breaches of confidentiality regarding self-disclosed personal information in the *tablet-based pre/post-test survey*. In order to minimize above risks associated with confidentiality:

1. No participant names will be collected and participants will be asked to mark X on the consent form to indicate their consent. This X will be used to ensure confidentiality. No identifying information will be collected (i.e. no full names, no date of birth).

2. Participants have the option to take a copy of the informed consent form, which will be the only evidence linking them to the study, or to view it when/if they choose by going to NEGES where there will be a copy of the informed consent available to view.

3. PHW trained in research and confidentiality issues will work on an individual basis with participants to administer the tablet-based surveys. Participants, regardless of literacy, will be supported and guided to fill out the tablet-based survey independently to maintain privacy if they wish.

4. PHW will be well trained in research ethics and interviewing techniques with special attention paid to confidentiality. PHW will also sign confidentiality agreements.

5. PHW will be assigned to work with participants who they do not have a personal or professional relationship with. The study coordinator will inform the participant that she can choose to work with a different PHW who she may have been assigned to work with for the pre/post test and the intervention.

Another social risk is that PHW who facilitates the *weekly women’s health discussions* cannot assure that all discussion members will keep all information provided in the discussion confidential or that discussion members may know one another.

In order to minimize the above risks:

1) Participants may skip any discussion questions, if they feel uncomfortable or if they do not wish to answer or participate in group activities. Additionally, the consent form offers the possibility of requesting to be part of a different group, which will be made available to the participant.

2) PHW facilitators will be well trained in research ethics and interviewing techniques. They will stress to other discussion participants the importance of keeping everything said during the discussion confidential. They will additionally indicate that confidentiality cannot be assured and that participants should refrain from saying anything that they would not be comfortable having repeated outside the discussion.

**7.2 Benefits**

There are no direct benefits to participants. However, as the study is designed to enhance understanding of HIV and STI knowledge, HIV and STI risk factors, depression, trauma, social support, and relationship power among women living in IDP camps in Leogane, Haiti, in order to inform health and mental health practice and policy, participants may benefit from knowing they have contributed to this knowledge.

Capacity building will be an important component of this study, providing peer health workers with training and short term employment, will hopefully enhance skills and research experience. Peer driven recruitment (PDR) has also been demonstrated to promote community engagement and knowledge dissemination of findings back to the community (Tiffany, 2006).

There is currently a dearth of HIV and STI intervention and education research focused on women in IDP camps, particularly in Haiti. There is also little known about the current state of mental health, coping and social support, and HIV and STI risk factors following the 2010 earthquake. Results will be disseminated to community-based agencies working with women, people living in IDP camps, people living with HIV, heath care practitioners and social workers. It is hoped that findings can be used as an evidence base to support policy and practice initiatives to promote health and well being for women living in IDP camps in Haiti. Additionally, the study is designed to highlight strengths and strategies for promoting mental health through examining social support and resilient coping.

**8. TIMELINE 2011-2012**

| **TASK** | **J** | **A** | **S** | **O** | **N** | **D** | **J** | **F** | **M** | **A** | **M** |
| --- | --- | --- | --- | --- | --- | --- | --- | --- | --- | --- | --- |
| Develop survey | **X** |  |  |  |  |  |  |  |  |  |  |
| Submit ethics | **X** |  |  |  |  |  |  |  |  |  |  |
| Develop training manual/videos |  | **X** | **X** | **X** |  |  |  |  |  |  |  |
| Train women as peer health workers |  |  |  |  | **X** |  |  |  |  |  |  |
| Study recruitment |  |  |  |  |  | **X** |  |  |  |  |  |
| Pre-test data collection |  |  |  |  |  |  | **X** |  |  |  |  |
| Weekly women’s health meetings |  |  |  |  |  |  |  | **X** | **X** |  |  |
| Post-test data collection |  |  |  |  |  |  |  |  | **X** | **X** |  |
| Community forum and knowledge translation |  |  |  |  |  |  |  |  |  |  | **X** |

**9. REFERENCES**

Beck, A.T., Guth, D., Steer, R.A., & Ball, R. (1997). Screening for major depressive disorders in medical inpatients with the beck depression inventory for primary care. *Behavioral Research Therapy, 35(8),* 785-791.

Braun, V. & Clarke, V. (2006) Using thematic analysis in psychology. *Qualitative Research in Psychology, 3,* 77-101.

Brewin, C.R., Rose, S., Andrews, B., Green, J., Tata, P., McEvedy, C., Turner, S. & Foa, E.B. (2002). Brief screening instrument for post-traumatic stress disorder. *British Journal of Psychiatry, 181,* 158-162.

Carey, M.P. & Schroder, K.E.E. (2002). Development and psychometric evaluation of the brief HIV knowledge questionnaire (HIV-KQ-18). *AIDS Education and Prevention, 14,* 174-184.

Charmaz, K. (2006). *Constructing grounded theory.* Thousand Oaks, California: Sage Publications Inc.

Dorjgochoo, T., Noel, F., Deschapms, M.M., Theodore, H., Dupont, W., Wright, P.F., Fitzgerald, D.W., Vermund, S.H. & Pape, J.W. (2009). Risk factors for HIV infection among Haitian adolescents and young adults seeking counseling and testing in Port-au-Prince. *Journal of Acquired Immune Deficiency Syndromes, 52,* 498-508.

Dunkle, K.L., Jewkes, R.K., Brown, H.C., Gray, G.E., McIntryre, J.A. & Harlow, S.D. (2004). Gender-based violence, relationship power, and risk of HIV infection in women attending antenatal clinics in South Africa. *Lancet, 363,* 1415-1421.

Field, A. (2009). *Discovering statistics using SPSS.* Third Edition. London: Sage Publications Inc.

Hampton, H. (2008). Innovative program offers HIV therapy to internally displaced persons in Uganda. *Journal of the American Medical Association, 300*(5), p. 493.

Inter-Agency Standing Committee (IASC). (2010). *Guidelines for addressing HIV in humanitarian settings*. Geneva, Switzerland: UNAIDS.

Internal Displacement Monitoring Centre (IDMC). (2011). *Internal displacement: global overview of trends and developments in 2010.* Geneva, Switzerland: Internal Displacement Monitoring Centre, Norweigan Refugee Council.

Ivers, L.C., Mukherjee, J.S., Leandre, F.R., Rigodon, J., Cullen, K.A. & Furin, J. (2010). South-south collaboration in scale-up of HIV care: building human capacity for care. *AIDS, 24*(S1), S73-S78.

Jaworski, B.C. & Carey, M.P. (2007). Development and psychometric evaluation of a self-administered questionnaire to measure knowledge of sexually transmitted diseases. *AIDS & Behavior, 11*, 557-574.

Khaw, A.J., Burkholder, B., Salama, P. & Dondero, T. (2000). HIV risk and prevention in emergency-affected populations: a review. *Disasters, 24*(3), 181-197.

MADRE. (2011). *One Year Update—Our Bodies Are Still Trembling: Haitian Women Continue to Fight Against Rape*. New York: Madre & City University of New York (CUNY) School of Law. Port-au-Prince, Haiti: Institute for Justice & Democracy in Haiti (IJDH).

Magnani, R., Sabin, K., Saidel, T. & Heckathorn, D. (2005). Review of sampling hard-to-reach and hidden populations for HIV surveillance. *AIDS, 19 (S2),* S67-S72.

Pulerwitz, J., Gortmaker, S.L. & DeJong, W. (2002). Measuring sexual relationship power in HIV/STD research. *Sex Roles, 42 (7/8),* 637-660.

Rujumba, J. & Kwiringira, J. (2010). Interface of culture, insecurity and HIV and AIDS: Lessons from displaced communities in Pader District, Northern Uganda. *Conflict and Health, 4*(18), 1-10.

Sinclair, V.G., & Wallston, K.A. (2004). The development and psychometric evaluation of the brief resilient coping scale. *Assessment, 11(1),* 94-101.

Tiffany, J.S. (2006). Respondent-driven sampling in participatory research contexts: Participant driven recruitment. *Journal of Urban Health: Bulletin of the New York Academy of Medicine, 83 (7),* 113-123.

UNAIDS. (2010). *2010 report on the global AIDS epidemic.* Geneva: Joint United Nations Programme on HIV/AIDS.

UNAIDS. (2011). AIDS Info: Haiti. Downloaded February 15, 2011 from: http://www.unaids.org/en/Regionscountries/Countries/Haiti/

UNAIDS. (2011b). *Progress in restoring access to HIV services in Haiti.* Downloaded February 15, 2011 from: <http://www.unaids.org/en/resources/presscentre/featurestories/2011/january/20110112haiti/>

UN Women (2011). *UN Women on the ground: Haiti’s women a year after the earthquake.* United Nations Entity for Gender Equality and the Empowerment of Women.

UNICEF. (2011). *Children in Haiti: one year after—the long road from relief to recovery.* Haiti Country Office: United Nations Children’s Fund.

Zimet, G.D., Dahlem, N.W., Zimet, S.G., & Farley, G.K. (1988). The multidimensional scale of perceived social support. *Journal of Personality Assessment, 52(1),* 30-41.

Zimet, G.D., Powell, S.S., Farley, G.K., Werkman, S., & Berkoff, K.A. (1990). Psychometric characteristics of the multidimensional scale of perceived social support. *Journal of Personality Assessment, 55(3&4),* 610-617.
